# Othello Syndrome Induced by Dopamine Agonists in Parkinson's Disease: A Case Report

**DOI:** 10.1155/crps/8517387

**Published:** 2025-09-05

**Authors:** Ghazi Uddin Ahmed, Haadi Uddin Ahmed, Ahmed Asad Raza, Abedin Samadi

**Affiliations:** ^1^Department of Medicine, Jinnah Sindh Medical University, Karachi, Pakistan; ^2^Department of Medicine, Kabul University of Medical Science, Kabul, Afghanistan

**Keywords:** delusional jealousy, dopamine agonist, othello syndrome, Parkinson's disease, ropinirole

## Abstract

**Background:** Othello syndrome (OS) is a rare psychiatric disorder characterized by delusional jealousy and unfounded suspicions of infidelity. It has been associated with neurological diseases such as Parkinson's disease (PD), particularly in patients receiving dopamine agonists (DAs).

**Case Presentation:** A 69-year-old man with a longstanding diagnosis of PD developed OS after transitioning from levodopa/carbidopa to ropinirole due to intolerance. Six months after initiating ropinirole, the patient began experiencing intense, delusional beliefs regarding his wife's fidelity, despite no cognitive decline or psychiatric history. The delusional symptoms significantly strained his marital relationship.

**Clinical Findings and Diagnostic Assessment:** Neurological examination remained consistent with PD, and no structural brain abnormalities were observed on magnetic resonance imaging (MRI). The temporal association between ropinirole use and symptom onset led to the diagnosis of ropinirole-induced OS. Ropinirole was gradually discontinued over 4 weeks, and quetiapine was introduced. The patient showed substantial improvement in psychiatric symptoms with resolution of delusional beliefs and restoration of spousal rapport.

**Conclusion:** This case highlights the potential for DAs, including ropinirole, to induce OS in PD patients. Clinicians should remain vigilant for psychiatric side effects during PD treatment and consider timely intervention, including medication adjustment and antipsychotic therapy, to prevent severe psychosocial consequences.

## 1. Introduction

Othello syndrome (OS) is a psychiatric condition characterized by intense, irrational jealousy in which a person becomes convinced of their partner's infidelity without any factual basis [1]. This delusional disorder has been observed alongside various neurological and psychiatric conditions, including cerebrovascular accidents, head injuries, brain neoplasms, degenerative brain diseases, central nervous system infections, demyelinating disorders, idiopathic normal pressure hydrocephalus, hormonal imbalances, and substance-induced psychosis [2]. Reports indicate that approximately 1.1% of hospitalized psychiatric patients exhibit delusional jealousy, with schizophrenia, delusional disorder, and chronic alcoholism being the most frequent underlying diagnoses [3]. Neurobiological studies suggest that OS, like other delusional syndromes, may involve impaired functioning of the right frontal lobe [4]. The extreme emotions and irrational beliefs associated with this condition can escalate into aggressive or even violent behavior, creating significant risks for both patients and their close relatives [5]. The syndrome derives its name from Shakespeare's Othello, in which the protagonist's baseless suspicions lead him to murder his wife, Desdemona [6].

While rare, OS has been documented in Parkinson's disease (PD) patients undergoing treatment with dopamine agonists (DAs) [7]. PD is a progressive neurodegenerative disorder primarily defined by motor impairments such as bradykinesia, rigidity, and tremor, resulting from dopaminergic neuron loss in the substantia nigra and the presence of Lewy bodies [8]. Clinical evidence suggests that all dopaminergic medications, including levodopa, ergot-derived DAs, and nonergot DAs, can contribute to the onset of OS, with no significant difference in risk among drug classes. A study of 563 nondemented PD patients identified six individuals who developed OS [9].

## 2. Case Presentation

The patient was a 69-year-old, right-handed, married man who had retired after working as an engineer for a private airline. He lived with his wife in an urban setting, while his only son resided abroad. Although father and son maintained occasional contact, the emotional distance and physical separation contributed to chronic feelings of loneliness and social isolation, which became more apparent as the patient's disease progressed. The patient's past medical history was significant only for PD, with no prior psychiatric illnesses, no cognitive impairment, and no history of substance use. The family history was negative for neurodegenerative or psychiatric disorders. The patient had no known allergies, and his socioeconomic status was stable, allowing reasonable access to medical care.

**Figure 1:**
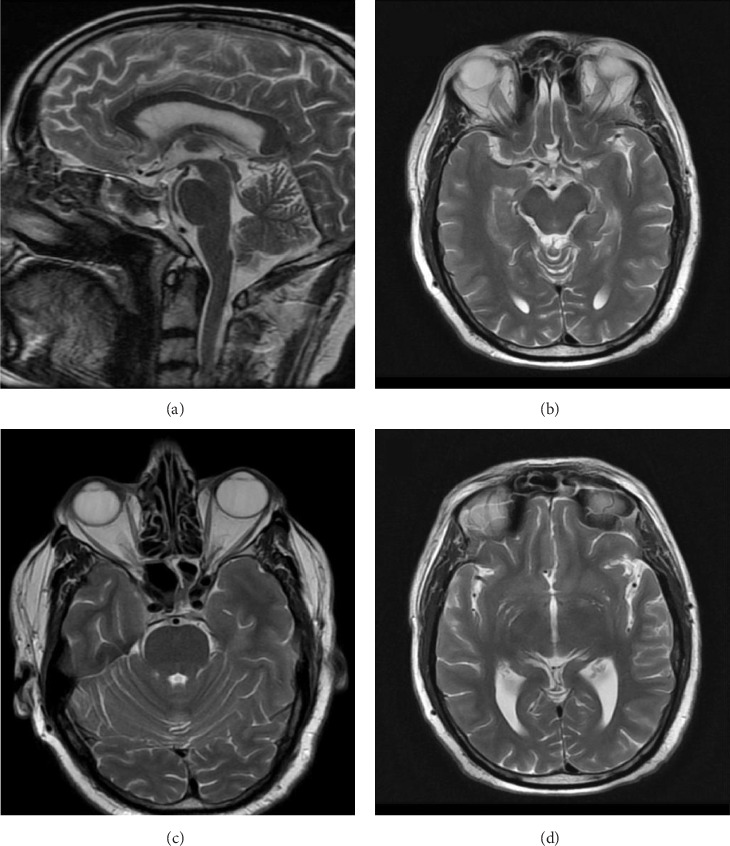
MRI brain scans of the patient (a) sagittal T2-weighted image showing the midbrain and brainstem with preserved pontine and cerebellar structures. (b) Axial T2-weighted image at the basal ganglia level, with no significant putaminal atrophy or hypointensity. (c) Axial T2-weighted image at the midbrain level, where substantia nigra changes may be seen in PD on advanced sequences. (d) Axial T2-weighted image showing mild cortical atrophy, which can be seen in advanced PD.

Eight years prior to writing this report, the patient reported to the outpatient department (OPD) with complaints of progressive bradykinesia, resting tremor, and limb rigidity, significantly impacting his daily activities. Clinical evaluation was consistent with a diagnosis of PD, supported by brain magnetic resonance imaging (MRI), which demonstrated no major structural abnormalities but subtle findings compatible with early-stage PD (Figure 1). Specifically, MRI brain scans revealed preserved midbrain and brainstem structures, no significant basal ganglia abnormalities, and mild cortical atrophy suggestive of early neurodegenerative changes.

Upon diagnosis, pharmacologic therapy was initiated with levodopa/carbidopa, amantadine, and rasagiline. Entacapone was not prescribed at the time due to its high cost and the lack of a locally available generic formulation. Although entacapone later became available in combination with levodopa/carbidopa, the patient continued with his original regimen. Anticholinergic agents were not used after the patient declined them following informed discussions regarding potential side effects such as cognitive decline and urinary retention.

Over the next several years, the patient adhered to his treatment plan with regular OPD visits. However, he began to experience intolerable side effects from levodopa/carbidopa, including visual hallucinations, persistent nausea, vomiting, and episodes of dizziness, which led to a significant decline in his quality of life. These adverse effects necessitated a gradual tapering and eventual discontinuation of levodopa/carbidopa therapy. Subsequently, the patient experienced worsening bradykinesia and rigidity, prompting the introduction of the DA ropinirole. Ropinirole was initiated at a low dose of 0.25 mg daily, with slow weekly increments to a maximum dose of 6 mg per day over a period of approximately 5–6 months.

Approximately 6 months after achieving the target ropinirole dose, the patient's wife reported new and concerning behavioral changes. The patient developed paranoid delusions, primarily centered around unfounded suspicions of his wife's infidelity. Initially intermittent and mild, these delusions escalated over 6 weeks, progressively straining their marital relationship. Despite these psychiatric symptoms, the patient demonstrated no signs of cognitive impairment; his Addenbrooke's Cognitive Examination-Revised (ACE-R) [10] score was 91, indicative of preserved cognitive function. Neurological examination remained consistent with PD without additional deficits. There was no prior psychiatric history in the patient or his immediate family to suggest a primary psychiatric disorder.

The clinical course of the patient, including major diagnostic and therapeutic interventions, is summarized in Table 1.

Based on the temporal association between the titration of ropinirole and the onset of psychosis, DA-induced psychosis was suspected. The decision was made to gradually taper and discontinue ropinirole over a 4-week period. During this time, the patient was admitted to the hospital for close inpatient monitoring to prevent a sudden deterioration in motor symptoms or psychiatric exacerbation. During hospitalization, following levodopa discontinuation, the patient's motor symptoms worsened markedly, with increased bradykinesia and rigidity, reduced ambulation capacity requiring assistance for short distances, and partial dependance on caregivers for basic activities of daily living. Clozapine was not considered for antipsychotic therapy due to its well-documented risk of agranulocytosis and the need for intensive hematological monitoring.

As part of his management plan, quetiapine was initiated at a low dose and gradually titrated upward to control psychotic symptoms without significantly worsening Parkinsonian motor symptoms. This was in alignment with current clinical practice, favoring quetiapine for managing PD psychosis due to its minimal extrapyramidal side effects. Following these interventions, the patient exhibited a remarkable clinical response. Within 2 weeks, the paranoid delusions had significantly abated, and his wife reported notable improvements in their interpersonal relationship and his overall demeanor.

During follow-up visits, the patient's psychiatric symptoms remained well controlled, although his Parkinsonian motor symptoms showed slow progression, consistent with the natural history of the disease. Two months after psychiatric stabilization, the patient was brought to the emergency department with acute respiratory distress, hypoxia, and reduced responsiveness. Despite aggressive medical management, including airway support, intravenous fluids, and empirical antibiotic therapy for suspected pneumonia, his condition deteriorated rapidly. He passed away within 48 h of admission. Based on clinical judgment, his death was presumed to be secondary to complications of advanced PD, possibly aspiration pneumonia, although no autopsy was performed at the family's request.

The patient's wife, when interviewed during bereavement follow-up, expressed appreciation for the brief period of emotional reconnection they were able to experience after the resolution of his psychotic symptoms. She emphasized that the improvement in his mental status had provided meaningful closure for their relationship during the patient's final months.

## 3. Discussion

OS is recognized as the paranoid delusion most frequently linked to suspicions of spousal infidelity. The exact pathophysiology of OS remains uncertain, though it is hypothesized that excessive dopamine activity in the mesolimbic pathways may contribute to psychotic symptoms by disrupting thought processes [11]. Research involving 116 PD patients indicated an OS prevalence of 5.2% [12].

Although OS typically manifests in middle-aged individuals, with younger age being a potential risk factor, the condition was observed in an elderly patient in this case. A study by Sabiry et al. [13] involving four OS patients reported an average onset age of approximately 50 years, usually developing 4–9 years after PD diagnosis in those taking DAs, with cognitive decline noted in half of the cases and symptom improvement following DA dosage reduction. However, unlike these findings, the patient discussed here exhibited intact cognitive function, as demonstrated by a normal ACE-R score, implying that OS can occur without cognitive deficits in certain PD patients [13].

OS rarely appears in isolation and is frequently accompanied by hallucinations unrelated to delusional jealousy. In PD patients, delusions combined with hallucinations may indicate a progression toward more severe psychotic symptoms, particularly when insight is lacking. Additionally, abnormal sexual or impulse control behaviors are more prevalent among patients on DA therapy, as seen in this case [14]. It is noteworthy that hallucinations and impulse control disorders, including pathological gambling, hypersexuality, and compulsive shopping, are far more frequently observed psychiatric side effects of dopaminergic therapy in PD than OS, which remains rare in comparison [15]. While all dopaminergic medications carry a risk of inducing OS and psychotic symptoms, no strong association has been established between specific dopaminergic drugs and OS onset, though studies on this remain limited [14].

The dopamine D3 receptor (DRD3) is believed to play a central role, given its involvement in reward processing, cravings, and emotional and cognitive functions [16]. Pramipexole, a potent DRD3 agonist, is the most probable trigger for OS, though other DAs like ropinirole and pergolide may also contribute due to their DRD3 receptor affinity [17].

Treatment for OS aligns with general psychotic disorder management strategies. The primary intervention involves tapering or discontinuing anti-Parkinsonian drugs, especially DAs, ideally substituting them with levodopa at equivalent doses. If delusional jealousy persists, second-generation antipsychotics such as clozapine or quetiapine may be added as supplementary therapy [18].

## 4. Conclusion

OS is a rare psychiatric disorder that is more common in patients with early disease onset and should systematically be scanned at each consultation to avoid dramatic marital distress and conflict. Once confirmed, the neurologist should initiate DA withdrawal and, if necessary, additional treatment with antipsychotics is recommended. Usage of DA is a risk factor for OS in patients with PD, regardless of the presence or absence of dementia.

## Figures and Tables

**Table 1:** Timeline of clinical events.

Timepoint	Clinical event
8 years prior	Diagnosis of Parkinson's disease; started on levodopa/carbidopa, amantadine, rasagiline.
5–6 years prior	Development of side effects from levodopa/carbidopa; medication gradually discontinued.
2 years prior	Initiation of ropinirole at 0.25 mg/day, with slow titration to 6 mg/day.
1.5 years prior	Emergence of paranoid delusions associated with ropinirole.
1.4 years prior	Tapering and discontinuation of ropinirole; initiation of quetiapine; psychiatric symptoms resolved.
1.2 years prior	Acute deterioration and death presumed secondary to advanced Parkinson's disease.

## Data Availability

The data supporting the findings of this study are available from the corresponding author upon reasonable request. Due to the nature of the study, patient confidentiality and ethical considerations prevent the public from sharing sensitive clinical data. All other data related to the study, including de-identified information, can be made available upon request, in accordance with the journal's data-sharing policies.
